# Resveratrol and curcumin enhance pancreatic β-cell function by inhibiting phosphodiesterase activity

**DOI:** 10.1530/JOE-14-0335

**Published:** 2014-11

**Authors:** Michael Rouse, Antoine Younès, Josephine M Egan

**Affiliations:** 1 Laboratory of Clinical Investigation, National Institute on Aging, Intramural Research Program, National Institutes of Health, 251 Bayview Blvd, Baltimore, Maryland, 21224, USA; 2 Laboratory of Cardiovascular Science, National Institute on Aging, Intramural Research Program, National Institutes of Health, 251 Bayview Blvd, Baltimore, Maryland, 21224, USA

**Keywords:** type 2 diabetes, resveratrol, curcumin, phosphodiesterase, β-cell

## Abstract

Resveratrol (RES) and curcumin (CUR) are polyphenols that are found in fruits and turmeric, and possess medicinal properties that are beneficial in various diseases, such as heart disease, cancer, and type 2 diabetes mellitus (T2DM). Results from recent studies have indicated that their therapeutic properties can be attributed to their anti-inflammatory effects. Owing to reports stating that they protect against β-cell dysfunction, we studied their mechanism(s) of action in β-cells. In T2DM, cAMP plays a critical role in glucose- and incretin-stimulated insulin secretion as well as overall pancreatic β-cell health. A potential therapeutic target in the management of T2DM lies in regulating the activity of phosphodiesterases (PDEs), which degrade cAMP. Both RES and CUR have been reported to act as PDE inhibitors in various cell types, but it remains unknown if they do so in pancreatic β-cells. In our current study, we found that both RES (0.1–10 μmol/l) and CUR (1–100 pmol/l)-regulated insulin secretion under glucose-stimulated conditions. Additionally, treating β-cell lines and human islets with these polyphenols led to increased intracellular cAMP levels in a manner similar to 3-isobutyl-1-methylxanthine, a classic PDE inhibitor. When we investigated the effects of RES and CUR on PDEs, we found that treatment significantly downregulated the mRNA expression of most of the 11 PDE isozymes, including *PDE3B*, *PDE8A*, and *PDE10A*, which have been linked previously to regulation of insulin secretion in islets. Furthermore, RES and CUR inhibited PDE activity in a dose-dependent manner in β-cell lines and human islets. Collectively, we demonstrate a novel role for natural-occurring polyphenols as PDE inhibitors that enhance pancreatic β-cell function.

## Introduction

Diabetes mellitus (DM) is a chronic metabolic disease that, in 2013, by global estimates has a worldwide prevalence of 382 million people. The number is expected to increase to 592 million by 2035, most having type 2 DM (T2DM; Guariguata *et al*. 2014). T2DM is generally associated with abdominal obesity and dyslipidemia, which can be brought about by calorie-dense diets and sedentary lifestyles (Mokdad *et al*. 2003, Samson & Garber 2014). Pancreatic β-cells are key players in the development of T2DM, as they are required to secrete increasing amounts of insulin so as to compensate for increasing insulin resistance. Consequently, the β-cells come under increasing metabolic stress and finally their function deteriorates (Marshak *et al*. 1999, Kahn 2003). Thus, it is important to find a means to preserve the health of β-cells.

Cyclic nucleotide phosphodiesterases (PDEs) belong to a class of enzymes that hydrolyze the phosphodiester bonds of cAMP and cGMP, thereby modulating various cellular signaling pathways. Thus far, eleven families of PDEs have been uncovered, which vary in their affinities for cAMP and cGMP as well as in expression levels within tissues (Cote *et al*. 2007). In fact, multiple PDEs along with their associated isozymes have been reported to be expressed at both mRNA and protein levels in rodent islets and β-cell lines (Härndahl *et al*. 2002, Cantin *et al*. 2007, Dov *et al*. 2008, Waddleton *et al*. 2008). Family-selective PDE inhibitors have proven to be very valuable tools for determining specific functions of selected PDEs and their roles in the developments in certain diseases. Several studies, for example, have demonstrated that inhibition of PDE3B (Härndahl *et al*. 2002, Waddleton *et al*. 2008), PDE8B (Dov *et al*. 2008), and PDE10A (Cantin *et al*. 2007) expression enhances β-cell function by promoting insulin secretion in response to glucose in rat β-cells.

Resveratrol (RES; 3,4′,5-trihydroxystilbene) is a polyphenolic compound found in grapes, berries, and peanuts. Reports of recent studies have described a number of health benefits that can be attributed to RES, such as its ability to serve as a chemopreventive agent by augmenting cAMP in human breast cancer cells (El-Mowafy & Alkhalaf 2003) and offering protection in cardiovascular disease through increased cGMP production in coronary arterial smooth muscle cells (El-Mowafy 2002). Furthermore, RES has been shown to possess therapeutic potential in the fight against obesity and T2DM (Beaudeux *et al*. 2010). In one study, RES was found to improve the health and survival of mice fed on a high-calorie diet (Baur *et al*. 2006). Results from another study indicated that RES treatment protected mice against diet-induced obesity and insulin resistance (Lagouge *et al*. 2006). The extent of beneficial effects of RES has also been tested in a number of diabetic models including streptozotocin (STZ), nicotinamide/STZ, and long-term high-fat diets (Su *et al*. 2006, Szkudelski & Szkudelska 2011, Fiori *et al*. 2013). Recent studies in humans have revealed that RES supplementation improved glycemic control, and insulin sensitivity, and reduced oxidative stress in T2DM patients (Kar *et al*. 2009, Brasnyó *et al*. 2011, Bhatt *et al*. 2012).

For centuries, turmeric has been used in traditional medicine to treat various ailments and diseases. Additionally, curcumin (CUR) supplementation has been noted as having many health benefits (Aggarwal *et al*. 2003). CUR, like RES, is a natural polyphenol that has shown great potential as a novel therapeutic agent due to its pharmacological safety and efficacy in treating a wide variety of human diseases. As an anti-oxidant, CUR has been shown to reverse STZ-induced hyperglycemia/glucose tolerance, hypoinsulinemia, and damage of pancreatic islets (El-Azab *et al*. 2011). CUR was also able to protect human islets from oxidative stress by increasing the mRNA and protein expression of heme oxygenase 1, glutathione, and NAD(P)H:quinone oxidoreductase 1 (Balamurugan *et al*. 2009). Results have also indicated that CUR treatment has anti-inflammatory properties in patients with inflammatory bowel diseases (Holt *et al*. 2005). More recently, CUR has emerged as a potential means to prevent and treat diabetes. This assertion comes after a 9-month study, involving a pre-diabetic population, which demonstrated that CUR treatment could not only lower HbA1c and homeostasis model assessment of insulin resistance (HOMA-IR) levels (a measure of insulin sensitivity), but also decelerate the deterioration of pre-diabetes to T2DM (Chuengsamarn *et al*. 2012). In addition, other studies have revealed CUR's ability to ameliorate hyperglycemic and hyperlipidemic conditions in diabetic animals (Arun & Nalini 2002, Hussain 2002, Pari & Murugan 2005). However, despite the wealth of health benefits provided by RES and CUR, particularly in the realm of diabetes, the molecular targets and mechanisms of action remain abstruse.

In the current study, we investigate the effects of RES and CUR on pancreatic β-cell function. We found that under both low- and high-glucose conditions, treatment with either RES or CUR led to a significant increase in insulin secretion in mouse β-Min6 cells as well as human islets. Treatment with these natural products also augmented intracellular levels of cAMP, an important second messenger in the insulin secretion pathway. We then developed a novel PDE activity assay so that we could directly assay PDE activity in β-cells. Our results indicate that RES and CUR enhance β-cell function by regulating PDE expression and activity, thus increasing intracellular cAMP levels and subsequent insulin secretion.

## Materials and methods

### Reagents

RES was purchased from Tocris Bioscience (Minneapolis, MN, USA). CUR, 3-isobutyl-1-methylxanthine (IBMX), DMSO, d-glucose, ATP assay mix, calmodulin from bovine heart, myokinase (adenylate kinase) from rabbit muscle, pyruvate kinase from rabbit muscle, cAMP, ATP, AMP, CTP, PEP, dithiothreitol (DTT), Protease Inhibitor Cocktail, and all other chemicals used in this study were obtained from Sigma.

### Cell culture

β-Min6 cells were maintained at 37 °C in an atmosphere of 5% CO_2_ in high glucose DMEM, which was supplemented with 15% heat-inactivated fetal bovine serum, 1% penicillin/streptomycin, and sodium pyruvate. Human pancreatic islet β-cells HP62, an epithelial human pancreatic islet cell line of insular origin generated by transfection of islet monolayer cultures with the plasmid pX8, which contains SV40 early region, were provided by Drs Marta Vives and Ricardo Pujol-Borrell. HP62 cells retain insulin production during the initial six passages and were maintained at 37 °C in an atmosphere of 5% CO_2_ in RPMI 1640 medium, which was supplemented with 10% heat-inactivated fetal bovine serum, 1% penicillin/streptomycin, 2 mmol/l glutamine, 0.5% transferrin, and 10 nmol/l hydrocortisone (Soldevila *et al*. 1991). Human islets were maintained at 37 °C in an atmosphere of 5% CO_2_ in CMRL medium consisting of 5.5 mmol/l glucose, 1% penicillin/streptomycin, 2 mmol/l glutamine, and 3% BSA. Stock Krebs buffer (pH 7.4) was saturated with 95% O_2_/5% CO_2_, and contained 137 mmol/l NaCl, 4.7 mmol/l KCl, 1.2 mmol/l KHPO_4_, 1.2 mmol/l MgSO_4_-7H_2_O, 2.5 mmol/l CaCl_2_-2H_2_O, and 25 mmol/l NaHCO_3_.

### Insulin secretion assay

β-Min6 cells (passages 9–12) were seeded at a density of 4×10^6^ cells/well in a 12-well plate for 24 h at 37 °C in an atmosphere of 5% CO_2_. Human islets were separated into 60 cm dishes containing 50 islets/dish. Cells were washed three times with glucose-free Krebs buffer, and then incubated in 0.05% BSA Krebs buffer (1 mmol/l glucose) for 1 h at 37 °C in an atmosphere of 5% CO_2_. Cells were again washed three times with glucose-free Krebs buffer. Afterwards, cells were cultured in 0.05% BSA Krebs buffer (1 or 25 mmol/l glucose) and treated with vehicle, IBMX, RES, or CUR at the indicated doses for 2 h. Supernatants were collected for insulin measurements using the Ultra-Sensitive Mouse Insulin ELISA Kit (Crystal Chemical, Inc., Downers Grove, IL, USA) for mouse or the Mercodia Insulin ELISA Kit (Mercodia, Winston Salem, NC, USA) for human cells, and performed according to the manufacturer's instructions.

### Intracellular cAMP assay

β-Min6 cells (passages 9–12) were seeded at a density of 4×10^6^ cells/well in a 12-well plate for 24 h at 37 °C in an atmosphere of 5% CO_2_. Mouse insulinoma cells were washed three times with glucose-free Krebs buffer before being placed in 0.05% BSA Krebs buffer (1 mmol/l glucose) for 1 h at 37 °C in an atmosphere of 5% CO_2_. After washing again with glucose-free Krebs buffer, cells were cultured in 0.05% BSA Krebs buffer (1 mmol/l glucose) and treated with vehicle, IBMX, RES, or CUR at the indicated doses. Primary human islets were plated at a concentration of 50 islets/60 mm dish in CMRL media (5 or 25 mmol/l glucose) containing 3% BSA and treated with RES or CUR at the indicated doses. Media were then removed and cells lysed using 0.1 mol/l HCl. Lysates were spun down to remove debris, while lysate supernatants were collected for intracellular cAMP measurements using a Direct cAMP ELISA Kit according to the manufacturer's instructions (ENZO Life Sciences, Farmingdale, NY, USA).

### Quantitative PCR

RNA was extracted using TRIzol (Invitrogen) and an RNeasy Mini Kit (Qiagen). RNA from mouse β-Min6 (passages 9–12) and human HP62 cell lines (passages 4–6) was converted into cDNA using qScript cDNA Supermix (Quanta Biosciences, Gaithersburg, MD, USA). RNA from human islets was converted into cDNA using a SuperScript III First-Strand Synthesis System (Invitrogen). PDE expression in β-Min6 and human HP62 cell lines as well as human islets was quantified using SYBR Green (Quanta Biosciences), and values were normalized to 18S (Ambion, Austin, TX, USA). Quantitative PCR was performed on an ABI Prism 7300 (Applied Biosystems) detection system. The mouse primers were: *Pde3b* (forward: 5′-AGTATCAGTAGCTTGATGGGTGC-3′ and reverse: 5′-CCCTTGTGAAGTTTTCGATCTCC-3′), *Pde8a* (forward: 5′-TGCAATTTGGCCCGATGAGAT-3′ and reverse: 5′-TGGAATCCGTTACACTGGCTA-3′), and *Pde10a* (forward: 5′-AGGATACGAATATGCAGGGAGT-3′ and reverse: 5′-CCGTCGGCTTTTGTGGCTAT-3′) (Integrated DNA Technologies, Coralville, IA, USA). The human primers were: PDE3B (forward: 5′-TTCAGGAGACCGTCGTTGC-3′ and reverse: 5′-TGACACCATATTGCGAGCCTC-3′), PDE8A (forward: 5′-AAAACCCCAACATCATGGCCT-3′ and reverse: 5′-CCTGAGTTTCAGTTGTGATCGC-3′), and PDE10A (forward: 5′-GAGACAACCAGCTACTCCTCT-3′ and reverse: 5′-ACAGGCTATTATTGCACTCTCCA-3′) (Integrated DNA Technologies).

### PDE activity assay

Mouse β-Min6 (passages 9–12) or human HP62 cells (passages 4–6) were seeded into 100 cm dishes at 37 °C in an atmosphere of 5% CO_2_ until 80% confluency was reached in a fresh culture medium. Human islets were provided by the National Institute of Diabetes and Digestive and Kidney Diseases-funded Integrated Islet Distribution Program at the City of Hope. For primary cultures, human islets were placed in 100 cm dishes containing 150 islets/dish. Cells were washed three times with glucose-free Krebs buffer and then incubated in 0.05% BSA Krebs buffer (1 mmol/l glucose) for 1 h at 37 °C in an atmosphere of 5% CO_2_. Cells were again washed three times with glucose-free Krebs buffer. Afterwards, β-Min6 and HP62 cells were cultured in 0.05% BSA Krebs buffer (1 or 25 mmol/l glucose) for 2 h, while primary human islets were cultured in 0.05% BSA Krebs buffer (5 or 25 mmol/l glucose) for 2 h. Then, cells were homogenized in cell lysis buffer containing 20 mmol/l HEPES (pH 7.4), 0.5 mmol/l EDTA, 2 mmol/l MgCl_2_, 0.1% Triton X-100, 0.5 mmol/l DTT, 1 mmol/l EGTA, and Protease Inhibitor Cocktail. Lysates were filtered on GE Healthcare (Pittsburgh, PA, USA) PD MidiTrap G-25 sample preparation columns (Fisher Scientific, Pittsburgh, PA, USA), and the protein concentration was determined by BCA Protein Assay (Pierce, Rockford, IL, USA). Assay buffers were spiked with vehicle, RES, or CUR as indicated before being added to cell lysates. Bioluminescence PDE activity assays were performed in 96-well plates (Opaque Proxiplate half-area microplates, Perkin Elmer, Waltham, MA, USA) using a Promega GloMax Multi-Detection System as described previously (Younès *et al*. 2011).

### Statistical analysis

Quantitative data are expressed as the mean±s.e.m. Differences between mean values were compared statistically by one-way ANOVA followed by the Bonferroni's *post hoc* comparison. A *P* value of <0.05 was considered statistically significant.

## Results

### RES and CUR enhance pancreatic β-cell function

**Figure 1:**
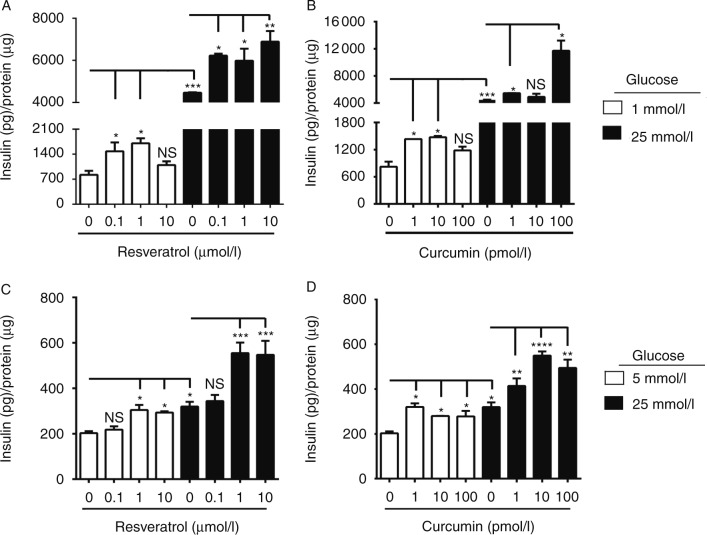
Resveratrol (RES) and curcumin (CUR) enhance insulin secretion in pancreatic β-cells. Mouse β-Min6 cells were treated with (A) RES or (B) CUR for 2 h under low- (1 mmol/l) or high- (25 mmol/l) glucose conditions. Primary human islets (*n*=2 donors) were incubated with (C) RES or (D) CUR for 2 h under low- (5 mmol/l) or high- (25 mmol/l) glucose conditions. Supernatants from triplicate samples were analyzed for insulin secretion (*P<0.05, **P<0.01, ***P<0.001, and ****P<0.0001). Data are representative of at least three independent experiments.

We treated β-Min6 cells with different doses of RES and CUR for 2 h. These doses have been reported to be biologically achievable based on bioavailability and pharmacokinetic studies in animals and humans (Shoba *et al*. 1998, Lao *et al*. 2006, Yoshino *et al*. 2012, Poulsen *et al*. 2013). In addition, cells were cultured under low- (1 mmol/l) and high- (25 mmol/l) glucose conditions. We found that RES (0.1–1 μmol/l) increased insulin secretion in a dose-dependent manner under low-glucose conditions (Fig. 1A). When cultured under high-glucose conditions, we observed that all three doses of RES (0.1–10 μmol/l) significantly increased insulin secretion compared with control. Similarly, CUR, albeit at significantly lower doses (1–10 pmol/l), markedly increases insulin secretion under low-glucose conditions (Fig. 1B). In addition, CUR (up to 100 pmol/l) greatly enhanced insulin secretion under high-glucose conditions compared with secretion from untreated cells. We then investigated whether these polyphenols could elicit similar effects in human islets. When examining the effects of the polyphenols on human islets, we found that slightly higher concentrations of RES (1–10 μmol/l) were required to significantly enhance insulin secretion compared with non-treated islets when cultured under low- (5 mmol/l) or high- (25 mmol/l) glucose conditions (Fig. 1C). CUR, on the other hand, was able to significantly augment insulin secretion compared with non-treated islets at a dose of 1–100 pmol/l (Fig. 1D). Treatment with either RES or CUR was found to maintain heightened insulin levels at 24 h; however, the combination RES+CUR either did not yield any additional benefits or reduced the beneficial effects observed with the individual treatments (Supplementary Figs 1 and 2, see section on supplementary data at the end of this article). These results indicate that individual treatment with natural polyphenols, such as RES and CUR, can positively influence rodent as well as human β-cell function.

**Figure 2:**
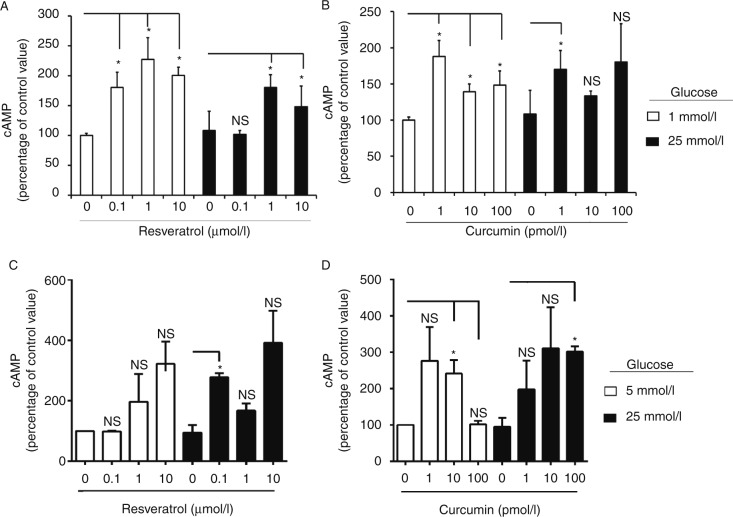
Resveratrol (RES) and curcumin (CUR) increase intracellular cAMP levels in β-cells. Mouse β-Min6 cells were treated with (A) RES or (B) CUR for 2 h under low- (1 mmol/l) or high- (25 mmol/l) glucose conditions. Primary human islets were treated with (C) RES or (D) CUR for 2 h under low- (5 mmol/l) or high- (25 mmol/l) glucose conditions (*n*=2 donors). Cells were lysed and assessed for intracellular cAMP levels after normalizing to protein content in triplicates (*P<0.05). Data are representative of at least three independent experiments.

We then investigated the effects of RES and CUR on intracellular β-cell events. After 2 h of culture under low-glucose conditions, RES almost doubled intracellular cAMP levels in β-Min6 cells compared with untreated cells (Fig. 2A) and when cells were cultured under high-glucose conditions, RES (1 and 10 μmol/l) markedly increased intracellular cAMP levels. CUR also substantially increased intracellular cAMP levels in β-Min6 cells under both low- and high-glucose conditions (Fig. 2B). When human islets were treated with RES or CUR, both polyphenols were able to increase intracellular levels (Fig. 2C and D). Again, the combination RES+CUR did not display an additive effect (Supplementary Fig. 3, see section on supplementary data given at the end of this article). These results indicate that RES and CUR probably enhance β-cell function under normal as well as diabetic conditions because of intracellular cAMP production and a consequent enhancement of insulin secretion.

**Figure 3:**
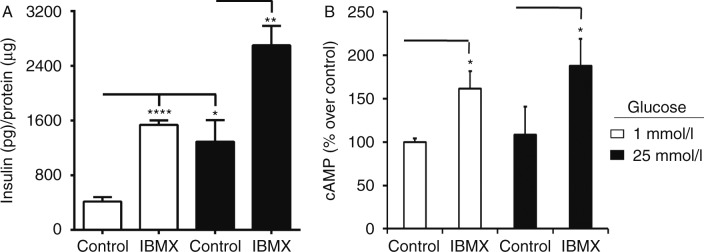
PDE blockade leads to enhanced insulin secretion and intracellular cAMP production. Mouse β-Min6 cells were treated with PDE inhibitor IBMX (50 μmol/l) for 2 h under low- (1 mmol/l) or high- (25 mmol/l) glucose conditions. Cells were examined for (A) insulin secretion and (B) intracellular cAMP levels (normalized to protein content; *P<0.05, **P<0.01, and ****P<0.0001). Samples were run in triplicate, and data are representative of at least three independent experiments.

PDEs belong to a class of enzymes capable of breaking the phosphodiester bonds that comprise second messenger molecules such as cAMP. Consequently, PDEs are considered important regulators of signal transduction of cAMP-mediated pathways, such as insulin secretion. To prove that inhibitors of PDEs augment cAMP levels and affect insulin secretion in our cell system, we treated β-Min6 cells with IBMX, a non-specific PDE inhibitor. We witnessed a significant rise in insulin secretion under low- and high-glucose conditions following IBMX (50 μmol/l) treatment (Fig. 3A). Subsequently, we also observed a substantial increase in intracellular cAMP levels compared with non-IBMX-treated cells (Fig. 3B). These results indicate that the blockade of PDEs enhances β-cell function through cAMP modulation.

### RES and CUR reduce *PDE* gene expression in β-cells

**Figure 4:**
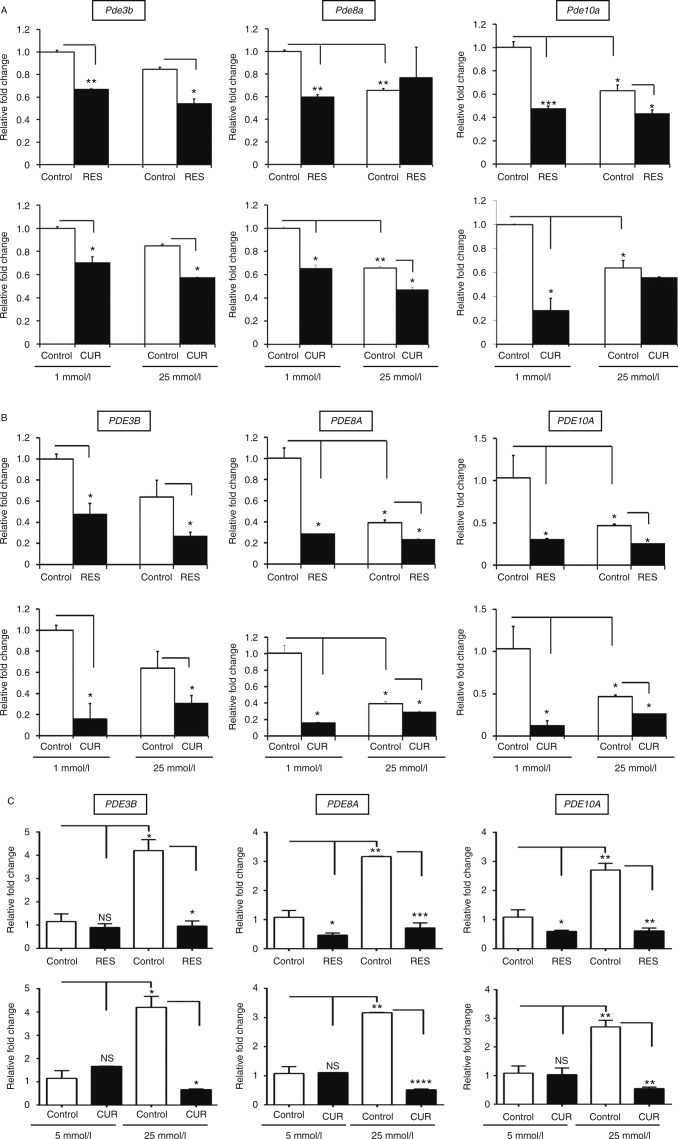
Resveratrol (RES) and curcumin (CUR) reduce PDE expression in β-cells. (A) Mouse β-Min6 cells and (B) human HP62 β-cells were incubated with vehicle, RES (0.1 μmol/l), or CUR (1 pmol/l) for 2 h under low- (1 mmol/l) or high- (25 mmol/l) glucose conditions. (C) Primary human islets (*n*=3 donors) were incubated with vehicle, RES (10 μmol/l), or CUR (100 pmol/l) for 2 h under low (5 mmol/l) or high (25 mmol/l) glucose conditions. Samples, ran in triplicate, were analyzed for relative *PDE* mRNA expression using quantitative RT-PCR and results are expressed as mean±s.e.m. (*P<0.05, **P<0.01, ***P<0.001, ****P<0.0001). Data are representative of at least three independent experiments.

We investigated the effects of RES and CUR on *PDE* gene expression and function to determine whether these polyphenolic compounds modulated PDEs in β-cells. We first examined the mRNA expression of known mouse *Pde* isoforms and established that a majority of them were downregulated following RES or CUR treatment (results not shown). As a result, we continued to focus primarily on three main *Pde* isoforms reported to act as critical regulators in the insulin secretion pathway: *Pde3b*, *Pde8a*, and *Pde10a* (Fig. 4A). Using the lowest effective dose, RES (0.1 μmol/l) significantly reduced the relative mRNA expression of *Pde3b*, *Pde8a*, and *Pde10a* in mouse β-Min6 cells cultured under low-glucose conditions. CUR (1 pmol/l) also decreased *Pde3b*, *Pde8a*, and *Pde10a* mRNA expression under low-glucose conditions. When the β-Min6 cells were cultured in a high-glucose environment, RES-treated cells had significantly lower expression levels of *Pde3b* and *Pde10a*, but there were no significant changes in *Pde8a*. Meanwhile, treatment with CUR under high-glucose conditions led to substantial decreases in *Pde3b* and *Pde8a* mRNA expression. CUR, however, did not appear to alter the expression of *Pde10a* in β-Min6 cells cultured under high-glucose conditions, indicating subtle differences in effects between CUR and RES.

Following our studies using the mouse cell line, we investigated whether RES and CUR had similar effects on *PDE* mRNA expression in human HP62 β-cells. RES treatment (0.1 μmol/l) extensively reduced mRNA expression of three *PDEs* important in the insulin signaling pathway under low-glucose conditions, and it also displayed similar efficacy under high-glucose conditions (Fig. 4B). In HP62 β-cells, CUR treatment (1 pmol/l) also decreased the expression of *PDE3B*, *PDE8A*, and *PDE10A* in both low and high glucose conditions. These results indicate that RES and CUR alter *PDE* expression independently from glucose in mouse as well as human β-cells.

To further investigate the translational applications of our study, we examined the effects of RES and CUR treatment on *PDE* mRNA expression in primary human islets (Fig. 4C). In these studies, RES treatment (10 μmol/l) was found to significantly diminish *PDE8A* and *PDE10A* expression under low-glucose conditions, while dramatically reducing expression of all three *PDE*s under high-glucose conditions. CUR (100 pmol/l), on the other hand, demonstrated no significant changes in the presence of low levels of glucose. However, CUR was able to substantially downregulate *PDE3B*, *PDE8A*, and *PDE10A* in the presence of high levels of glucose. It is important to note that although higher doses of RES and CUR might be needed for treating human islets compared with insulinoma cell lines due to the heterogenous composition of islets, we observed similar therapeutic effects on *PDE* expression.

### RES and CUR impede PDE activity in pancreatic β-cells

To directly study the ability of RES and CUR to act as PDE inhibitors in pancreatic β-cells, we developed an assay to directly measure the kinetics of PDE activity. In short, the PDE activity assay measures the degradation of cAMP to 5′-AMP by PDEs, thus levels of AMP increase at a directly proportional rate to the level of PDE activity within the system.

**Figure 5:**
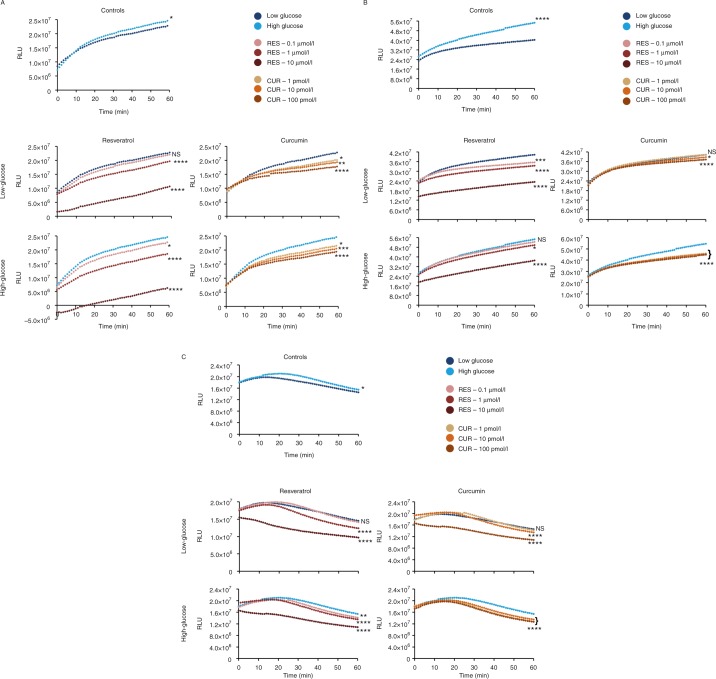
Resveratrol (RES) and curcumin (CUR) impede PDE activity in pancreatic β-cells. (A) Mouse β-Min6 cells and (B) human HP62 β-cells were cultured for 2 h under low- (1 mmol/l) or high- (25 mmol/l) glucose conditions. (C) Primary human islets (*n*=2 donors) were cultured for 2 h under low- (5 mmol/l) or high- (25 mmol/l) glucose conditions. Cell lysates were treated with vehicle, RES, or CUR and analyzed for relative PDE activity using a bioluminescence reaction assay. Samples were run in triplicates and the mean was plotted (*P<0.05, **P<0.01, ***P<0.001, and ****P<0.0001). Data are representative of at least three independent experiments.

In the current study, cell lysates were collected from β-Min6 cells cultured for 2 h under low- and high-glucose conditions. Control lysates were passed through a G-25 sample preparation column and subjected to an enzymatic reaction to assess PDE activity. During the course of the study, lysates from cells cultured under high-glucose conditions displayed a slightly higher PDE activity compared with those cultured under low-glucose conditions (Fig. 5A). When various doses of RES (0.1–10 μmol/l) were added to low-glucose lysates, RES was found to reduce PDE activity in a dose-dependent manner. The effects of RES appeared to be even more marked in the high-glucose lysates, illustrating that 10 μmol/l RES was most efficient at lowering PDE activity. When lysates of β-Min6 cells were exposed to CUR, PDE activity was decreased to similar degrees with each dose of CUR (1–100 pmol/l) under both low- and high-glucose conditions.

Lysates from HP62 cells, under high-glucose conditions, had considerably elevated levels of PDE activity compared with lysates under low-glucose conditions (Fig. 5B). In lysates from cells cultured under low glucose conditions, RES lowered PDE activity in a dose-dependent manner. In lysates under high-glucose conditions, increasing concentration of RES also led to a decreased PDE activity. CUR under low-glucose conditions had little to no effect on PDE activity. Conversely, HP62 cells exposed to CUR (1–100 pmol/l) in the presence of high glucose levels exhibited marked reductions in PDE activity.

Finally, we measured PDE activity in human islets. In the process, we did observe a slightly modified profile of the PDE activity curve due to the heterogeneity of islets and multiple cell types expressing PDEs compared with the homogenous β-cell lines. However, islets exposed to high levels of glucose continue to exhibit increased PDE activity compared with those exposed to low levels of glucose (Fig. 5C). Although 0.1 μmol/l RES did not appear to alter PDE activity in low-glucose lysates, at 1–10 μmol/l it starkly inhibited PDE activity. In lysates under high-glucose conditions, increasing RES concentrations significantly reduced PDE activity. CUR (1 pmol/l) had minimal effect, whereas 10 and 100 pmol/l caused a notable decrease in PDE activity in lysates under low-glucose conditions, while under high-glucose conditions CUR at all concentrations tested was a powerful inhibitor of PDE activity. Collectively, these findings prove that RES and CUR directly impede PDE activity in pancreatic β-cells, and that this mechanism of action is conserved across species.

## Discussion

In the current study, we explored the therapeutic prowess of natural products, specifically RES and CUR, in an attempt to enhance β-cell function. Previous animal models and human clinical trials have reported that oral administration of RES (5 mg–5 g; Carrizzo *et al*. 2013, Trimmers *et al*. 2013) or CUR (80 mg–6 g; Fan *et al*. 2013, Meng *et al*. 2013) given as a single dose or daily for up to 12 months to diabetics is able to reduce blood glucose and improve insulin sensitivity. Our results demonstrate the ability of RES and CUR to promote β-cell function across species by acting as direct PDE inhibitors in β-cells and islets. We found that treatment of mouse and human β-cells with RES and CUR resulted in a substantial reduction of PDE expression, particularly *PDE3B*, *PDE8A*, and *PDE10A*, which are known to be important in insulin signaling. Furthermore, RES and CUR treatment demonstrated a profound ability to directly inhibit PDE activity in β-cells as well as islets. As a result of PDE inhibition, RES and CUR treatment prevented the degradation of cAMP, thus leading to an increase in its intracellular levels. Subsequently, this allowed for activation of cAMP-dependent signaling pathways, thereby augmenting insulin secretion and β-cell function.

Results from several studies have indicated that naturally occurring polyphenols RES and CUR have shown promise in the realm of diabetes. Previously, results from our laboratory indicated that long-term RES supplementation prevented decreased expression of the essential β-cell transcription factors forkhead box protein O1 (*FOXO1*), *NKX6-1*, *NKX2-2*, and *PDX1* in rhesus monkeys given a high-fat high-sugar diet. In addition, we observed a similar type of protection upon RES treatment in human islets cultured under high-glucose+palmitate conditions (Fiori *et al*. 2013). In STZ-induced diabetic rats, CUR has demonstrated protection by displaying anti-oxidant and hypoglycemic properties (Hussain 2002, Pari & Murugan 2005), while RES also reduced the elevated levels of blood glucose as well as albumin, urea, and creatinine (Soufi *et al*. 2012). Other studies have demonstrated that mice given a high-fat diet display obesity, hyperglycemia, and insulin resistance, which were reversed upon treatment with either RES or CUR (Baur *et al*. 2006, Shao *et al*. 2012). RES treatment has also been shown to prevent age-related decreases in insulin sensitivity in primates, such as gray mouse lemur (*Microcebus murinus*; Marchal *et al*. 2012). Moreover, RES and CUR treatment have been shown to be effective in patients with T2DM by improving insulin sensitivity, and decreasing fasting blood glucose, HbA1c, triglyceride levels, and body weight (Brasnyó *et al*. 2011, Chuengsamarn *et al*. 2012, Kumar & Joghee 2013, Movahed *et al*. 2013). In the current study, we demonstrated that RES and CUR are able to significantly promote insulin secretion in mouse β-cell lines as well as human islets. The ability to enhance β-cell function was found to occur under both low- and high-glucose-stimulated conditions within a matter of hours.

The beneficial health benefits observed after RES and CUR supplementation have been attributed to their anti-inflammatory and anti-oxidant properties and even their ability to mimic caloric restriction. While many studies have explored the effects of RES and CUR on hyperglycemia as a whole, there remains limited evidence regarding their influence on β-cells. Therefore, we investigated and established the effects of RES and CUR treatment on the enhancement of β-cell function through an alternative and novel mechanism; they are direct PDE inhibitors. While the concept of RES and CUR acting as PDE inhibitors is a relatively new development, there is a growing amount of evidence that advocate the idea. One study, for example, revealed that RES dynamically increased cAMP levels in myotubes, thus triggering downstream effectors, such as PKA and AMPK. Additionally, the surge of cAMP was attributed to RES acting as a competitive and non-selective inhibitor of multiple PDEs (Park *et al*. 2012) and not as a result of activating adenylate cyclase (Gerhart-Hines *et al*. 2011). Similarly, CUR exhibited inhibitory properties on multiple PDE isozymes to elicit an anti-cancer and anti-proliferative effect in melanoma cells (Abusnina *et al*. 2011). These reports corroborate our findings and the newfound aptitude of RES and CUR as PDE inhibitors in various tissue types.

PDEs, which belong to a family of enzymes that degrade cAMP and cGMP (Soderling & Beavo 2000), serve as novel drug targets for treating various diseases, such as heart failure, depression, asthma, inflammation, and erectile dysfunction (Mehats *et al*. 2002, Rotella 2002). With regards to diabetes and insulin resistance, several PDEs have been noted to play important roles in regulating glucose tolerance through the modulation of cAMP-dependent processes. Rolipram, a selective PDE4 inhibitor, activated AMPK in myotubes, thus improving glucose tolerance in obese mice (Park *et al*. 2012). Previous studies have revealed that not only does isozyme PDE3B reside within insulin granules, but also that it mediates the acute first phase and the second sustained phase of insulin secretion (Walz *et al*. 2007). Moreover, the effects of PDE3B on β-cell function were further strengthened when the overexpression of PDE3B in mice led to impaired glucose-stimulated insulin secretion, glucose tolerance, and enhanced sensitivity to high-fat-diet-induced insulin resistance (Härndahl *et al*. 2002, 2004, Walz *et al*. 2006, 2007). In addition, blockade of PDE3B (Walz *et al*. 2007), PDE8B (Dov *et al*. 2008), and PDE10A (Cantin *et al*. 2007) expression and/or activity in pancreatic islets led to a significant increase in insulin secretion. Furthermore, in a study investigating diet-induced obesity, researchers found that inhibition of PDE10A, either through genetic deletion or pharmacological blockade, substantially increased weight loss and insulin sensitivity, while reducing adiposity in mice fed on a Western-style diet (Nawrocki *et al*. 2014).

Based on our observations, we are the first, to our knowledge, to show that treatment with RES or CUR leads to significant inhibition of PDE expression and activity in β-cells, thereby reducing the degradation of intracellular cAMP. In addition, individual treatment with RES or CUR markedly enhances β-cell function by triggering robust insulin secretion under low- and high-glucose conditions, whereas the combination RES+CUR hindered their beneficial effects. These findings indicate that RES and CUR could be acting through similar signaling pathways and may compete with each other for common substrates, such as PDE isozymes. Given the acute response of β-cells to these polyphenols, it would also be of interest to study the long-term effects on β-cell function following chronic treatment with RES or CUR. Similarly, as results from previous studies have indicated, PDEs play an important role in the regulation and development of T2DM and serve as critical therapeutic targets. Thus, it would be very informative to examine *PDE* expression levels as well as activity *in vivo* upon supplementation with RES or CUR. The strengths of our study are that multiple cell lines and isolated islets displayed similar improvements in function upon RES or CUR treatment, and, for the first time, to our knowledge, we show that we can directly measure, in real time, PDE activity in islets using our newly developed assay. Overall, the use of RES and CUR continues to show great therapeutic potential for enhancing β-cell function and mitigating the development of T2DM.

## Supplementary data

This is linked to the online version of the paper at http://dx.doi.org/10.1530/JOE-14-0335.

## Author contribution statement

M R designed, developed, and performed experiments, analyzed data, and wrote the manuscript. A Y designed and developed PDE activity assays in β-cells. M R and J M E were responsible for procuring human islets from the Integrated Islet Distribution Program and for the treatment of human islets. J M E contributed to the design of experiments, interpretation of data, and writing of the manuscript. All authors edited and reviewed the manuscript. J M E is the guarantor of this work and, as such, had full access to all the data in the study and takes responsibility for the integrity of the data and the accuracy of the data analysis.

## Supplementary Material

Supplementary Figure

## References

[bib1] Abusnina A, Keravis T, Yougbaré I, Bronner C, Lugnier C (2011). Anti-proliferative effect of curcumin on melanoma cells is mediated by PDE1A inhibition that regulates the epigenetic integrator UHRF1. Molecular Nutrition & Food Research.

[bib2] Aggarwal BB, Kumar A, Bharti AC (2003). Anticancer potential of curcumin: preclinical and clinical studies. Anticancer Research.

[bib3] Arun N, Nalini N (2002). Efficacy of turmeric on blood sugar and polyol pathway in diabetic albino rats. Plant Foods for Human Nutrition.

[bib4] Balamurugan AN, Akhov L, Selvaraj G, Pugazhenthi S (2009). Induction of antioxidant enzymes by curcumin and its analogues in human islets: implications in transplantation. Pancreas.

[bib5] Baur JA, Pearson KJ, Price NL, Jamieson HA, Lerin C, Kalra A, Prabhu VV, Allard JS, Lopez-Lluch G, Lewis K (2006). Resveratrol improves health and survival of mice on a high-calorie diet. Nature.

[bib6] Beaudeux JL, Nivet-Antoine V, Giral P (2010). Resveratrol: a relevant pharmacological approach for the treatment of metabolic syndrome?. Current Opinion in Clinical Nutrition and Metabolic Care.

[bib8] Bhatt JK, Thomas S, Nanjan MJ (2012). Resveratrol supplementation improves glycemic control in type 2 diabetes mellitus. Nutrition Research.

[bib9] Brasnyó P, Molnár GA, Mohás M, Markó L, Laczy B, Cseh J, Mikolás E, Szijártó IA, Mérei A, Halmai R (2011). Resveratrol improves insulin sensitivity, reduces oxidative stress and activates the Akt pathway in type 2 diabetic patients. British Journal of Nutrition.

[bib10] Cantin LD, Magnuson S, Gunn D, Barucci N, Breuhaus M, Bullock WH, Burke J, Claus TH, Daly M, Decarr L (2007). PDE-10A inhibitors as insulin secretagogues. Bioorganic & Medicinal Chemistry Letters.

[bib11] Carrizzo A, Forte M, Damato A, Trimarco V, Salzano F, Bartolo M, Maciag A, Puca AA, Vecchione C (2013). Antioxidant effects of resveratrol in cardiovascular, cerebral and metabolic diseases. Food and Chemical Toxicology.

[bib12] Chuengsamarn S, Rattanamongkolgul S, Luechapudiporn R, Phisalaphong C, Jirawatnotai S (2012). Curcumin extract for prevention of type 2 diabetes. Diabetes Care.

[bib7] Cote RH 2007 Photoreceptor phosphodiesterase (PDE6): A G-protein-activated PDE regulating visual excitation in rod and cone photoreceptor cells. In *Cyclic Nucleotide Phosphodiesterases in Health and Disease*, 1st Edn, Ch 8, pp 165–194. Eds J Beavo, S Francis & M Houslay. Boca Raton, FL: CRC Press.

[bib13] Dov A, Abramovitch E, Warwar N, Nesher R (2008). Diminished phosphodiesterase-8B potentiates biphasic insulin response to glucose. Endocrinology.

[bib14] El-Azab MF, Attia FM, El-Mowafy AM (2011). Novel role of curcumin combined with bone marrow transplantation in reversing experimental diabetes: effects on pancreatic islet regeneration oxidative stress, and inflammatory cytokines. European Journal of Pharmacology.

[bib15] El-Mowafy AM (2002). Resveratrol activates membrane-bound guanylyl cyclase in coronary arterial smooth muscle: a novel signaling mechanism in support of coronary protection. Biochemical and Biophysical Research Communications.

[bib16] El-Mowafy AM, Alkhalaf M (2003). Resveratrol activates adenylyl-cyclase in human breast cancer cells: a novel, estrogen receptor-independent cytostatic mechanism. Carcinogenesis.

[bib17] Fan X, Zhang C, Liu D, Yan J, Liang H (2013). The clinical applications of curcumin: current state and the future. Current Pharmaceutical Design.

[bib18] Fiori JL, Shin YK, Kim W, Krzysik-Walker SM, González-Mariscal I, Carlson OD, Sanghvi M, Moaddel R, Farhang K, Gadkaree SK (2013). Resveratrol prevents β-cell dedifferentiation in nonhuman primates given a high-fat/high-sugar diet. Diabetes.

[bib19] Gerhart-Hines Z, Dominy JE, Blättler SM, Jedrychowski MP, Banks AS, Lim JH, Chim H, Gygi SP, Puigserver P (2011). The cAMP/PKA pathway rapidly activates SIRT1 to promote fatty acid oxidation independently of changes in NAD^+^. Molecular Cell.

[bib20] Guariguata L, Whiting DR, Hambleton I, Beagley J, Linnenkamp U, Shaw JE (2014). Global estimates of diabetes prevalence for 2013 and projections for 2035. Diabetes Research and Clinical Practice.

[bib21] Härndahl L, Jing XJ, Ivarsson R, Degerman E, Ahrén B, Manganiello VC, Renström E, Holst LS (2002). Important role of phosphodiesterase 3B for the stimulatory action of cAMP on pancreatic β-cell exocytosis and release of insulin. Journal of Biological Chemistry.

[bib22] Härndahl L, Jing XJ, Ivarsson R, Degerman E, Ahrén B, Manganiello VC, Renström E, Holst LS (2004). β-Cell-targeted overexpression of phosphodiesterase 3B in mice causes impaired insulin secretion, glucose intolerance, and deranged islet morphology. Journal of Biological Chemistry.

[bib23] Holt PR, Katz S, Kirshoff R (2005). Curcumin therapy in inflammatory bowel disease: a pilot study. Digestive Diseases and Sciences.

[bib24] Hussain HE (2002). Hypoglycemic, hypolipidemic, and antioxidant properties of combination of curcumin from *Curcuma longa*, Linn, and partially purified product from *Abroma augusta*, Linn. in streptozotocin induced diabetes. Indian Journal of Clinical Biochemistry.

[bib25] Kahn SE (2003). The relative contribution of insulin resistance and β cell dysfunction to the pathophysiology of type 2 diabetes. Diabetologia.

[bib26] Kar P, Laight D, Rooprai HK, Shaw KM, Cummings M (2009). Effects of grape seed extract in type 2 diabetic subjects at high cardiovascular risk: a double blind randomized placebo controlled trial examining metabolic markers, vascular tone, inflammation, oxidative stress and insulin sensitivity. Diabetic Medicine.

[bib27] Kumar BJ, Joghee NM (2013). Resveratrol supplementation in patients with type 2 diabetes mellitus: a prospective, open label, randomized controlled trial. International Research Journal of Pharmacy.

[bib28] Lagouge M, Argmann C, Gerhart-Hines Z, Meziane H, Lerin C, Daussin F, Messadeq N, Milne J, Lambert P, Elliott P (2006). Resveratrol improves mitochondrial function and protects against metabolic disease by activating SIRT1 and PGC-1α. Cell.

[bib29] Lao CD, Ruffin MT, Normolle D, Heath DD, Murray SI, Bailey JM, Boggs ME, Crowell J, Rock CL, Brenner DE (2006). Dose escalation of a curcuminoid formulation. BMC Complementary and Alternative Medicine.

[bib30] Marchal J, Blanc S, Epelbaum J, Aujard F, Pifferi F (2012). Effects of chronic calorie restriction or dietary resveratrol supplementation on insulin sensitivity markers in a primate, *Microcebus murinus*. PLoS ONE.

[bib31] Marshak S, Leibowitz G, Bertuzzi F, Socci C, Kaiser N, Gross DJ, Cerasi E, Melloul D (1999). Impaired β-cell functions induced by chronic exposure of cultured human pancreatic islets to high glucose. Diabetes.

[bib32] Mehats C, Andersen CB, Filopanti M, Jin SL, Conti M (2002). Cyclic nucleotide phosphodiesterases and their role in endocrine cell signaling. Trends in Endocrinology and Metabolism.

[bib33] Meng B, Li J, Cao H (2013). Antioxidant and antiinflammatory activities of curcumin on diabetes mellitus and its complications. Current Pharmaceutical Design.

[bib34] Mokdad AH, Ford ES, Bowman BA, Dietz WH, Vinicor F, Bales VS, Marks JS (2003). Prevalence of obesity, diabetes, and obesity-related health risk factors, 2001. Journal of the American Medical Association.

[bib35] Movahed A, Nabipour I, Lieben Louis X, Thandapilly SJ, Yu L, Kalantarhormozi M, Rekabpour SJ, Netticadan T (2013). Antihyperglycemic effects of short term resveratrol supplementation in type 2 diabetic patients. Evidence-Based Complementary and Alternative Medicine.

[bib36] Nawrocki AR, Rodriguez CG, Toolan DM, Price O, Henry M, Forrest G, Szeto D, Keohane CA, Pan Y, Smith KM (2014). Genetic deletion and pharmacological inhibition of phosphodiesterase 10A protects mice from diet-induced obesity and insulin resistance. Diabetes.

[bib37] Pari L, Murugan P (2005). Effect of tetrahydrocurcumin on blood glucose, plasma insulin and hepatic key enzymes in streptozotocin induced diabetic rats. Journal of Basic and Clinical Physiology and Pharmacology.

[bib38] Park SJ, Ahmad F, Philp A, Baar K, Williams T, Luo H, Ke H, Rehmann H, Taussig R, Brown AL (2012). Resveratrol ameliorates aging-related metabolic phenotypes by inhibiting cAMP phosphodiesterases. Cell.

[bib39] Poulsen MM, Vestergaard PF, Clasen BF, Radko Y, Christensen LP, Stødkilde-Jørgensen H, Møller N, Jessen N, Pedersen SB, Jørgensen JO (2013). High-dose resveratrol supplementation in obese men: an investigator-initiated, randomized, placebo-controlled clinical trial of substrate metabolism, insulin sensitivity, and body composition. Diabetes.

[bib40] Rotella DP (2002). Phosphodiesterase 5 inhibitors: current status and potential applications. Nature Reviews. Drug Discovery.

[bib41] Samson SL, Garber AJ (2014). Metabolic syndrome. Endocrinology and Metabolism Clinics of North America.

[bib42] Shao W, Yu Z, Chiang Y, Yang Y, Chai T, Foltz W, Lu H, Fantus IG, Jin T (2012). Curcumin prevents high fat diet induced insulin resistance and obesity via attenuating lipogenesis in liver and inflammatory pathway in adipocytes. PLoS ONE.

[bib43] Shoba G, Joy D, Joseph T, Majeed M, Rajendran R, Srinivas PS (1998). Influence of piperine on the pharmacokinetics of curcumin in animals and human volunteers. Planta Medica.

[bib44] Soderling SH, Beavo JA (2000). Regulation of cAMP and cGMP signaling: new phosphodiesterases and new functions. Current Opinion in Cell Biology.

[bib45] Soldevila G, Buscema M, Marini V, Mirakian R, Deuss V, Sutton R, James R, Lake S, Robertson P, Pujol-Borrell R (1991). Transfection with SV40 genes of human pancreatic endocrine cells. Journal of Autoimmunity.

[bib46] Soufi FG, Sheervalilou R, Vardiani M, Khalili M, Alipour MR (2012). Chronic resveratrol administration has beneficial effects in experimental model of type 2 diabetic rats. Endocrine Regulations.

[bib47] Su HC, Hung LM, Chen JK (2006). Resveratrol, a red wine antioxidant, possesses an insulin-like effect in streptozotocin-induced diabetic rats. American Journal of Physiology. Endocrinology and Metabolism.

[bib48] Szkudelski T, Szkudelska K (2011). Anti-diabetic effects of resveratrol. Annals of the New York Academy of Sciences.

[bib49] Trimmers S, Hesselink MK, Schrauwen P (2013). Therapeutic potential of resveratrol in obesity and type 2 diabetes: new avenues for health benefits?. Annals of the New York Academy of Sciences.

[bib50] Waddleton D, Wu W, Feng Y, Thompson C, Wu M, Zhou YP, Howard A, Thornberry N, Li J, Mancini JA (2008). Phosphodiesterase 3 and 4 comprise the major cAMP metabolizing enzymes responsible for insulin secretion in INS-1 (832/13) cells and rat islets. Biochemical Pharmacology.

[bib51] Walz HA, Härndahl L, Wierup N, Zmuda-Trzebiatowska E, Svennelid F, Manganiello VC, Ploug T, Sundler F, Degerman E, Ahrén B (2006). Early and rapid development of insulin resistance, islet dysfunction and glucose intolerance after high-fat feeding in mice overexpressing phosphodiesterases 3B. Journal of Endocrinology.

[bib52] Walz HA, Wierup N, Vikman J, Manganiello VC, Degerman E, Eliasson L, Holst LS (2007). β-Cell PDE3B regulates Ca^2+^-stimulated exocytosis of insulin. Cellular Signalling.

[bib53] Yoshino J, Conte C, Fontana L, Mittendorfer B, Imai S, Schechtman KB, Gu C, Kunz I, Rossi Fanelli F, Patterson BW (2012). Resveratrol supplementation does not improve metabolic function in nonobese women with normal glucose tolerance. Cell Metabolism.

[bib54] Younès A, Lukyanenko YO, Lyashkov AE, Lakatta EG, Sollott SJ (2011). A bioluminescence method for direct measurement of phosphodiesterase activity. Analytical Biochemistry.

