# Supplementary material for: Resveratrol and curcumin enhance pancreatic β-cell function by inhibiting phosphodiesterase activity
Source: J Endocrinol. 2014 Nov;223(2):107–17. doi: 10.1530/JOE-14-0335 (PMC4191183; doi:10.1530/JOE-14-0335)
Supplement: Supplementary Figure [file supp_223_2_107__index.html]

Supplementary Figure 

# Resveratrol and curcumin enhance pancreatic β-cell function by inhibiting phosphodiesterase activity

## Supplementary Figure

**Files in this Data Supplement:**

- Supplementary Figure 1 - *Resveratrol and curcumin enhance insulin secretion in mouse β-Min6 insulinoma cells*. Mouse β-Min6 cells were treated with RES, CUR, or RES+CUR for 24 hrs under low (1 mmol L-1) or high (25 mmol L-1) glucose conditions at the indicated doses. Supernatants from triplicate samples were analyzed for insulin secretion upon normalizing to protein (\* p < 0.05, \*\*\*\* p < 0.0001). Data is representative from at least three independent experiments. (PDF 558 KB)
- Supplementary Figure 2 - *Resveratrol and curcumin enhance insulin secretion in human pancreatic islets*. Primary human islets (n = 2 donor) were treated with RES, CUR, or RES+CUR for 2 and 24 hrs under low (5 mmol L-1) or high (25 mmol L-1) glucose conditions at the indicated doses. Supernatants from triplicate samples were analyzed for insulin secretion upon normalizing to protein (\* p < 0.05, \*\* p < 0.01, \*\*\*\* p < 0.0001). Data is representative from at least three independent experiments. (PDF 723 KB)
- Supplementary Figure 3 - *Effects of resveratrol and curcumin on intracellular cAMP production in human pancreatic islets*. Primary human islets (n = 2) were treated with RES, CUR, or RES+CUR for 2 hrs under low (5 mmol L-1) or high (25 mmol L-1) glucose conditions at the indicated doses. Cells were lysed and assessed for intracellular cAMP levels after normalizing to protein content in triplicates (\* p < 0.05). Data is representative from at least three independent experiments. (PDF 512 KB)
