# Supplementary figures and images for: Resveratrol and curcumin enhance pancreatic β-cell function by inhibiting phosphodiesterase activity
Source: J Endocrinol. 2014 Nov;223(2):107–17. doi: 10.1530/JOE-14-0335 (PMC4191183; doi:10.1530/JOE-14-0335)

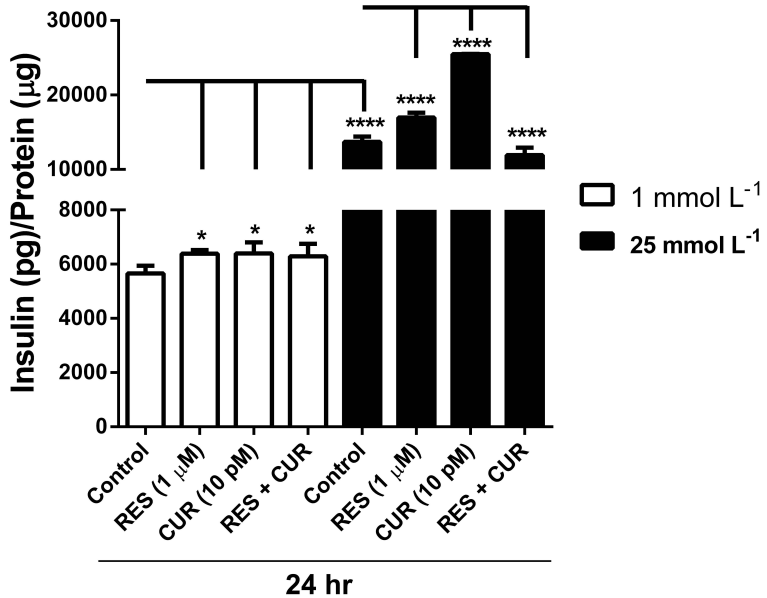

Supplement: Supplementary Figure [file supp_223.2.107_Supplementary_figure_1.pdf]

**A**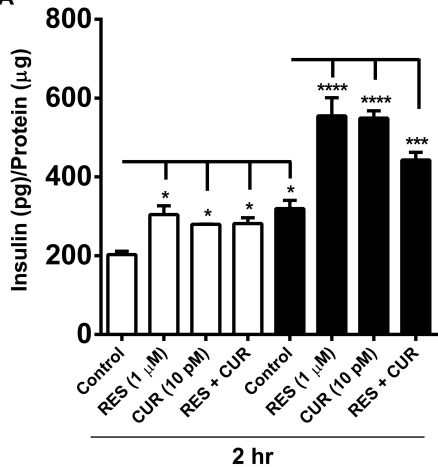**B**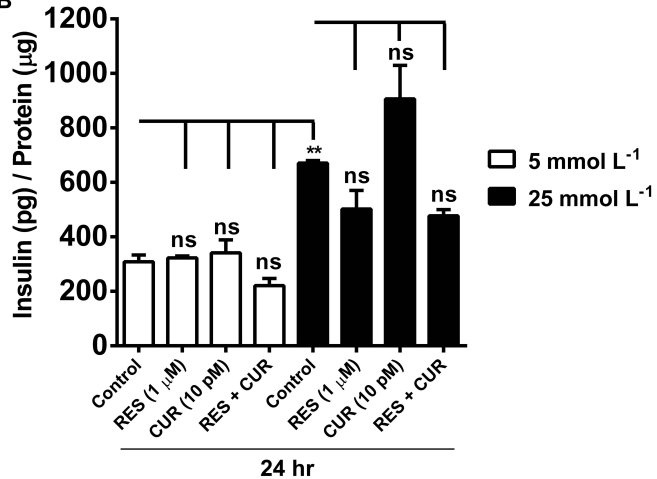

Supplement: Supplementary Figure [file supp_223.2.107_Supplementary_figure_2.pdf]

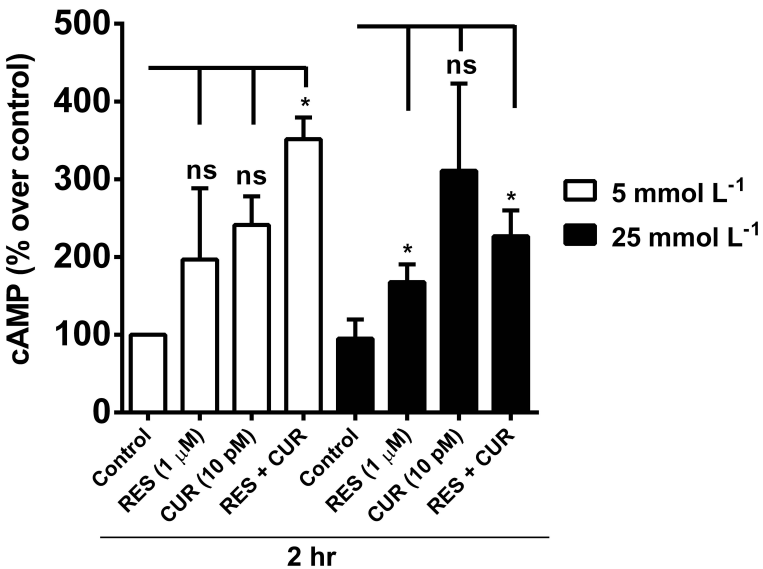

Supplement: Supplementary Figure [file supp_223.2.107_Supplementary_figure_3.pdf]
